# Glue Strips Measurement and Breakage Detection Based on YOLOv11 and Pixel Geometric Analysis

**DOI:** 10.3390/s25247624

**Published:** 2025-12-16

**Authors:** Yukai Lu, Xihang Li, Jingran Kang, Shusheng Xiong, Shaopeng Zhu

**Affiliations:** 1 Power Machinery & Vehicular Engineering Institute, Zhejiang University, Hangzhou 310014, China; yukai.lu@geely.com (Y.L.); xiongss@zju.edu.cn (S.X.); 2 Zhejiang Geely Automobile Co., Ltd., Hangzhou 310051, China; 3 College of Mechanical and Automotive Engineering, Ningbo University of Technology, Ningbo 315336, China; 4 Geely Automobile Research Institute (Ningbo) Co., Ltd., Ningbo 315336, China; jingran.kang@geely.com; 5 Longquan Industry Innovation Research Institute, Longquan 323700, China

**Keywords:** YOLOv11, pixel geometric analysis, battery pack glue, dimension measurement, glue breakage detection

## Abstract

With the rapid development of the new energy vehicle industry, the quality control of battery pack glue application processes has become a critical factor in ensuring the sealing, insulation, and structural stability of the battery. However, existing detection methods face numerous challenges in complex industrial environments, such as metal reflections, interference from heating film grids, inconsistent orientations of glue strips, and the difficulty of accurately segmenting elongated targets, leading to insufficient precision and robustness in glue dimension measurement and glue break detection. To address these challenges, this paper proposes a battery pack glue application detection method that integrates the YOLOv11 deep learning model with pixel-level geometric analysis. The method first uses YOLOv11 to precisely extract the glue region and identify and block the heating film interference area. Glue strips orientation correction and image normalization are performed through adaptive binarization and Hough transformation. Next, high-precision pixel-level measurement of glue strip width and length is achieved by combining connected component analysis and multi-line statistical strategies. Finally, glue break and wire drawing defects are reliably detected based on image slicing and pixel ratio analysis. Experimental results show that the average measurement errors in glue strip width and length are only 1.5% and 2.3%, respectively, with a 100% accuracy rate in glue break detection, significantly outperforming traditional vision methods and mainstream instance segmentation models. Ablation experiments further validate the effectiveness and synergy of the modules. This study provides a high-precision and robust automated detection solution for glue application processes in complex industrial scenarios, with significant engineering application value.

## 1. Introduction

**Figure 1:**
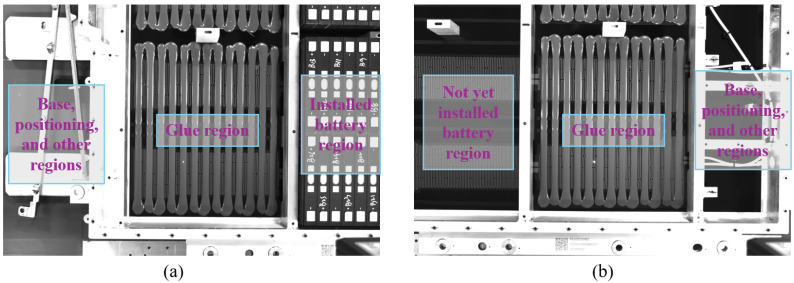
Glue process detection environment for new energy vehicle battery packs. (**a**) Example 1 of the glue process environment; (**b**) Example 2 of the glue process environment.

With the rapid development of the new energy vehicle industry, the reliability and efficiency of the manufacturing process for battery packs, as the core components of electric vehicles, have become key bottlenecks limiting the industrial scale-up [1,2]. Among these processes, the glue process of the battery pack is crucial for ensuring the sealing, insulation, and structural stability between the battery module and its shell. This directly affects the waterproofing, thermal management, and mechanical strength of the battery pack [3,4]. However, detecting the glue process faces challenges from complex environmental factors, as shown in Figure 1. These factors include base positioning, the glue region, and the installed/uninstalled battery regions, all of which may introduce interference (such as heating film grid patterns, metal reflections, and glue strip layout differences), making the implementation of detection methods more difficult.

Currently, battery pack glue detection mainly relies on manual visual detection and traditional machine vision methods. However, both methods have significant limitations. Although manual visual detection can judge the glue situation to a certain extent, it depends on the experience of the operator and is subject to subjective judgment and visual fatigue. Additionally, it is difficult to achieve micron-level size quantification, making it unable to meet the high-end manufacturing requirements for consistency [5]. Traditional machine vision methods, such as edge detection-based Canny algorithms and threshold-based binarization methods, have automated parts of the process. However, in complex glue scenarios, they are subject to multiple interference factors, such as the overlapping of heating film grid patterns with the glue strip color, blurred edges due to metal reflections on the base, and varying glue strip orientations and layouts for different models. This results in poor performance in interference suppression and scene adaptability [6,7]. Although object detection techniques represented by the YOLO series have achieved remarkable progress in the field of industrial vision in recent years, they still exhibit evident limitations in the specific scenario of glue application inspection for new energy vehicle battery packs. On the one hand, glue strips are typical slender targets with weak features at the head and tail regions as well as dense spacing, resulting in persistent deficiencies in existing segmentation models—particularly in instance segmentation of glue strips and contour extraction accuracy in the head and tail areas [8,9]. On the other hand, the segmentation results produced by deep learning models lack effective integration with pixel-level geometric analysis, making it difficult to achieve precise measurement of glue strip width and length, which in turn leads to considerable systematic errors in length measurement and glue breakage judgment [10,11]. Consequently, current methods still fall short of the industrial-grade requirements for high precision, high consistency, and strong robustness demanded by the battery pack glue application process.

To solve these problems, we propose a method for measuring the glue dimensions and detecting glue breakage in battery packs by combining the YOLOv11 deep learning model with pixel geometric analysis. First, the YOLOv11 model is used to detect and extract the glue region from input images. To ensure measurement consistency, the YOLOv11-seg model detects the orientation of the glue strips and corrects the image rotation to keep the glue strips in a vertical orientation, thus avoiding measurement errors due to changes in the glue strips orientation. For image interference, the YOLOv11 model first identifies and fills the heating film interference regions. Then, based on the detected glue strip mask’s pixel gray values, the binarization threshold is automatically set to adaptively distinguish between the glue strip and the background. Finally, combining pixel geometric analysis algorithms, the width and length of the glue strips are accurately measured, and an image slicing and pixel ratio analysis method is used to detect glue breakage and stretching defects, ensuring high robustness in complex environments. The main contributions of this paper are summarized as follows:

1. A glue detection framework integrating the YOLOv11 deep learning model and pixel geometric analysis is proposed. This framework significantly enhances the measurement accuracy and defect detection robustness in complex industrial environments, providing an efficient automated solution for zero-defect production of new energy vehicle battery packs.
2. The adaptive binarization, Hough line transformation correction, and image slicing analysis are fused to accurately measure the pixel-level width and length of glue strips and detect glue breakage/stretching defects, overcoming challenges from complex interference and small-sample defects, and improving detection robustness and generalization.

## 2. Related Work

### 2.1. Traditional Visual Detection Methods

Traditional visual inspection methods primarily rely on classical image processing techniques, including edge detection (such as Canny and its variants), thresholding, binarization, contour extraction, and subsequent pixel-level geometric analysis. These methods can achieve high accuracy in dimension measurement and defect detection in scenarios with simple backgrounds and regular workpiece structures [12,13]. Notable works include the following: Wang et al. [14], who addressed the issue of uneven brightness in lithium battery coatings using background reconstruction and an improved Canny algorithm; Guo et al. [15], who significantly improved the speed and accuracy of brake disk scratch detection using bidirectional connected Canny operators; and Pawat et al. [16] as well as Mikel et al. [17], who achieved high-precision angle measurement and adaptive edge finishing without deep learning. Additionally, Liu et al. [18], Xie et al. [19], Cheng et al. [20], and Ding et al. [21] further demonstrated that through geometric operations such as connected component analysis, minimum bounding rectangles, convex hulls, Hough transforms, contour fitting, and least squares methods, high-precision and robust detection of key dimensions and defects (e.g., fractures, notches, burrs, foreign objects, and shape deviations) can be achieved in complex industrial environments and under small-sample conditions, without the need for extensive labeled data [22,23].

However, in the battery pack glue inspection scenario, challenges such as strong metal reflections, heating film grid interference, uneven lighting, and subtle color differences between the glue and background lead to difficulties. Traditional edge detection methods often generate false edges, binarization thresholds are difficult to adapt, and geometric analysis is severely affected by noise, ultimately resulting in a significant decline in the robustness and measurement accuracy of these methods.

### 2.2. Deep Learning Visual Detection Methods

In recent years, deep learning techniques, particularly convolutional neural networks, have significantly advanced defect detection in the field of object detection. In particular, the YOLO (You Only Look Once) series of object detection models, through end-to-end training, can efficiently and accurately identify part defects [24,25]. For instance, YOLO-FIX [26] integrates deformable large kernel attention and Mamba-like linear attention mechanisms along with a multi-scale feature fusion module to effectively solve the complex shapes, background interference, and lighting changes in mobile phone frame glue line detection. YOLOv8n-SSE [27] introduces the SSE attention mechanism and WIoU loss function in automotive glue defect detection, which improves accuracy and detection speed compared to the original YOLOv8n, meeting real-time detection requirements. Chen et al. [28] propose a defect detection system based on automatic microscopic vision and the deep learning algorithm LBG-YOLO, which introduces a lightweight multi-scale convolutional module, a bidirectional feature pyramid network, and a global dependence coordinate attention mechanism to achieve real-time, high-precision defect detection for Micro-LEDs. ST-YOLO [29] improves detection accuracy and speed, while reducing model parameters, significantly optimizing photovoltaic defect detection performance by introducing the C2f-SCconv convolution module and the Triplet Attention mechanism. RDD-YOLO [30] improves the accuracy and speed of steel surface defect detection by introducing Res2Net [31], a dual-feature pyramid network, and a decoupled head. Although novel object detection models such as YOLOv11 exhibit strong adaptability in complex backgrounds and can effectively extract glue regions and suppress environmental interference, there are still challenges in handling small targets (e.g., thin glue strips), particularly in precise glue region segmentation and micro-sample detection. In this paper, based on the detection results of YOLOv11, we further integrate pixel-level geometric analysis, which significantly improves the accuracy and robustness of glue strip dimension measurement and defect detection.

## 3. Method

**Figure 2:**
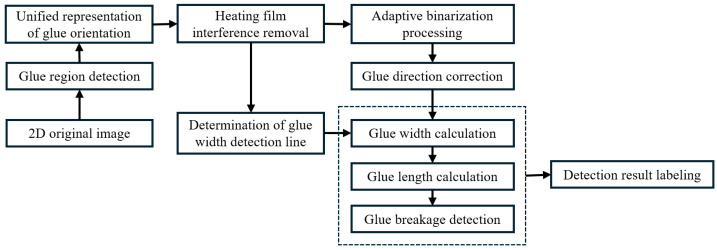
Workflow of battery pack glue strip dimension measurement and breakage detection method.

This paper proposes an automated detection method for the glue quality of new energy vehicle battery packs, combining deep learning with traditional image processing techniques. The method aims to efficiently and accurately address key issues in the glue process, such as dimension measurement (width, length) and defect detection (breakage or stringing). The core innovation of this work lies in using deep learning to achieve intelligent region extraction and interference suppression, as well as combining robust geometric analysis algorithms to achieve pixel-level parameter quantification. The algorithm workflow is shown in Figure 2. Next, we will detail the implementation process of each module.

### 3.1. Image Acquisition and Glue Region Extraction

In this study, a high-resolution industrial camera (resolution ≥ 1920 × 1080 pixels) is used to capture 2D images for quality detection of the glue on new energy vehicle battery packs. The camera is fixed above the glue station, at a distance of 150–200 cm from the glue surface, ensuring that the entire glue region is covered. To minimize environmental light interference, the image acquisition environment is equipped with uniform LED lighting (500–1000 lux), which helps to reduce the impact of reflections and shadows on image quality. Each image is captured once after the completion of the glue process, with images saved in BMP format at a standardized resolution of 1920 × 1080 pixels.

**Figure 3:**
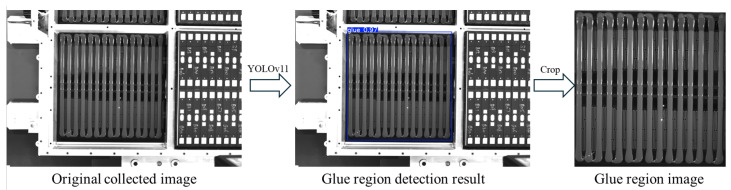
Schematic diagram of glue region detection.

Since the raw images contain a large amount of irrelevant information, such as the base, battery, cables, etc., directly extracting the glue strip status from these images would be severely interfered with, affecting detection accuracy and robustness. Therefore, we first trained a network based on the YOLOv11 model for detecting the glue region. By extracting the glue region from the image, we reduce interference, as shown in Figure 3.

### 3.2. Uniform Orientation Representation of Glue Strips

The direction of glue strips varies across different vehicle models, which complicates subsequent measurements of glue width and length. Therefore, to enhance the consistency of dimension measurement and glue breakage detection, we standardize the orientation of the glue strips, ensuring that the strips always maintain a vertical position. To achieve this, we design a glue strip orientation determination method based on the YOLOv11-seg model.

**Figure 4:**
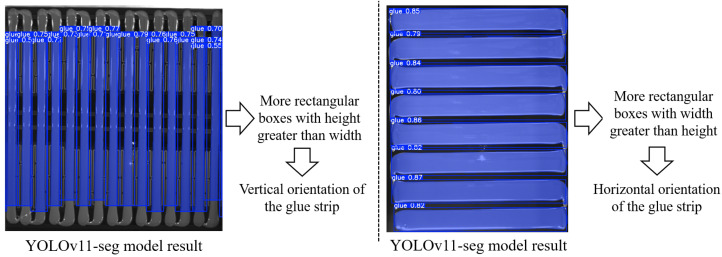
Schematic diagram of glue strip orientation determination.

First, the YOLOv11-seg model is used to extract the glue strip pixels within the glue region and mark them with bounding boxes. Next, the aspect ratio (h/w) of all bounding boxes is analyzed, and the number of bounding boxes with height greater than width is counted. If this number exceeds half of the total, the glue direction is judged to be vertical; otherwise, it is considered horizontal. If the direction is determined to be horizontal, the image is rotated 90° clockwise using OpenCV’s cv2.rotate function to ensure that the glue strip consistently maintains a vertical orientation. The rotated glue region image is denoted as *I*_glue_rotated_, as shown in Figure 4.

In practical experiments, we found that the YOLOv11-seg model faces some challenges when extracting the head and tail regions of the glue strip. This is because the head and tail portions of the glue strips are relatively small and lack distinct boundary or texture features, making it difficult for the model to accurately identify them. Furthermore, due to the limited number of samples, the model has certain limitations in learning small targets, particularly in image edge regions, where insufficient contextual information is available to support precise detection. Therefore, we further process the binarized image of the glue region and compute the relevant information of the glue strips, thereby improving detection accuracy.

### 3.3. Removal of Heating Film Interference and Adaptive Binarization

In some vehicle models, the bottom surface of the battery pack contains a heating film used for thermal management. The fine grid-like or linear texture on its surface, which is similar in color to the glue, can easily form high-frequency noise in the image. During the binarization stage, this may be mistakenly identified as part of the glue strip or cause edge discontinuities, severely affecting subsequent measurement accuracy.

To address this issue, we propose a heating film fine-line region detection algorithm based on YOLOv11. The specific steps are as follows: First, the YOLOv11 object detection module is used to identify the heating film fine-line regions in the image. After identification, the identified region is filled with black pixels to eliminate its interference with the subsequent analysis. Next, for adaptive binarization, we use the average grayscale value of the glue region pixels extracted by the YOLOv11-seg model, denoted as $P_{m}$. We empirically determine the binarization threshold as 0.85 times this value (i.e., $\tau =0.85P_{m}$) and apply it to the rotated glue region image *I*_glue_rotated_. This operation effectively separates the glue strips (foreground, assigned a value of 255) from the background (assigned a value of 0).

**Figure 5:**
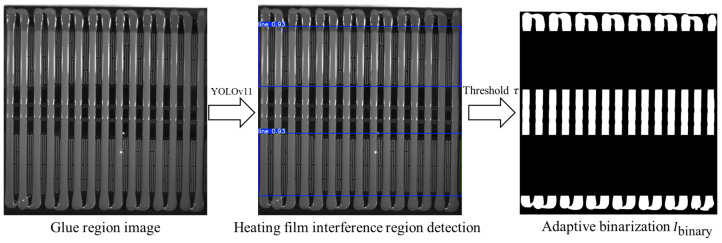
Identification of heating film interference regions and adaptive binarization.

To further remove small noise in the binarized image, morphological closing operations are applied (using a rectangular structuring element with a 5 × 5 kernel) to smooth the edges of the glue strips and fill in small holes. The final processed binarized image is denoted as $I_{\mathrm{binary}}$. As shown in Figure 5, the processed image effectively removes interference, improving the accuracy and robustness of subsequent analysis.

### 3.4. Glue Strip Direction Correction

**Figure 6:**
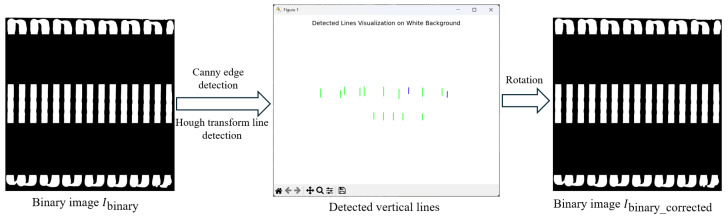
Schematic diagram of rotation correction. The green and blue lines represent the detected lines, with the blue line showing minimal deviation from the vertical direction.

Due to the deviation in the camera installation position and the inaccurate positioning of the battery pack base, the direction of the glue strips may not be perfectly vertical, which can affect the subsequent measurement of the glue strip’s width and length. Therefore, it is necessary to correct the direction of the glue strips to ensure they are strictly vertical. To achieve this, we propose a correction method based on image processing, as shown in Figure 6. The specific steps are as follows.

First, Canny edge detection is applied to the binarized image $I_{\mathrm{binary}}$ to extract the edges in the image. Then, the Hough Line Transform is used to process the edge image and detect the straight lines in the image. Each detected line can be represented in polar coordinates (ρ,θ), where ρ is the distance from the origin to the line, and θ is the angle between the line and the x-axis. Next, the lines that are close to the vertical direction are selected, with their angles θ satisfying $85^{\circ }\leq \theta \leq 105^{\circ }$. For the detected line segments, short lines are discarded, and the remaining lines are typically the edges of the glue strips.

Let the tilt angle of the *i*-th line be $\alpha_{i}$. The average angle $\alpha_{\mathrm{avg}}$ of all valid lines can be calculated using the following formula:

(1) $$\alpha_{\mathrm{avg}}=\frac{1}{n}\sum_{i=1}^{n}\alpha_{i}$$

where *n* is the number of valid lines.

Next, the calculated average tilt angle $\alpha_{\mathrm{avg}}$ is used to generate a rotation matrix. To correct the image’s tilt to vertical, we calculate the deviation $\Delta \alpha =90^{\circ }-\alpha_{\mathrm{avg}}$, and apply the rotation matrix to correct the image. The rotation matrix Ψ is calculated as follows:

(2) $$\Psi =\left[\begin{array}{ccc} \cos(\Delta \alpha ) & -\sin(\Delta \alpha ) & \left(1-\cos\left(\Delta \alpha \right)\right)\cdot c_{x}+\sin\left(\Delta \alpha \right)\cdot c_{y}\\ \sin(\Delta \alpha ) & \cos(\Delta \alpha ) & \left(1-\cos\left(\Delta \alpha \right)\right)\cdot c_{y}-\sin\left(\Delta \alpha \right)\cdot c_{x}\\ 0 & 0 & 1 \end{array}\right]$$

where $\left(c_{x},c_{y}\right)$ is the center of rotation in the image.

Using the rotation matrix Ψ, the corrected binarized image *I*_binary_corrected_ is obtained. The specific rotation operation is performed through affine transformation, and the calculation process is as follows:

(3) I binary_corrected=warpAffineIbinary,Ψ,(w,h)

where warpAffine(·) is an image transformation function that transforms the image according to the given rotation matrix Ψ, and *w* and *h* are the width and height of the image. This transformation maps each pixel in the image so that the direction of the glue strip is aligned close to vertical.

### 3.5. Glue Width Detection Line Region Determination

To avoid the influence of image edges or interference on the measurement of glue strip width, this method dynamically determines the measurement range of the glue region, with the following specific rules.

When no heating film fine-line interference exists in the image, the measurement region is selected from the central part of the image’s height *h*, specifically from 0.15*h* to 0.85*h*, covering 70% of the vertical height in the middle of the image. Then, detection lines are chosen at intervals of 10 pixels to measure the glue strip width. This strategy effectively avoids the upper and lower edge regions of the image, reducing the impact of external interference on measurement accuracy.

When a single heating film interference region exists in the image, the measurement region is dynamically adjusted based on the bounding box of the interference region. The interference region is identified using the YOLOv11 object detection module, which outputs bounding box coordinates $\left(x_{\min},y_{\min},x_{\max},y_{\max}\right)$, where $y_{\min}$ and $y_{\max}$ represent the upper and lower boundaries of the interference region (denoted as $y_{1}$ and $y_{2}$, respectively). In this case, the measurement region is divided into upper and lower parts. For the upper region, the center line of the measurement region is positioned at the midpoint between the upper boundary of the interference region $y_{1}$ and the top of the image y=0, i.e., $y_{\mathrm{center}\;}=y_{1}/2$. The upper and lower boundaries of the measurement region are extended 70% of the distance from the center line upwards and downwards, respectively, as follows:

(4) $$y_{\mathrm{upper}\;}=y_{\mathrm{center}\;}-0.35\times y_{\mathrm{center}}\;,\;y_{\mathrm{lower}\;}=y_{\mathrm{center}\;}+0.35\times y_{\mathrm{center}\;}$$

where $y_{\mathrm{upper}}$ and $y_{\mathrm{lower}}$ are the upper and lower boundaries, respectively.

The measurement range for the lower region is similar to that of the upper region and is not further detailed here. Then, within the detection region, detection lines are chosen at intervals of 10 pixels for measuring the glue strip width.

**Figure 7:**
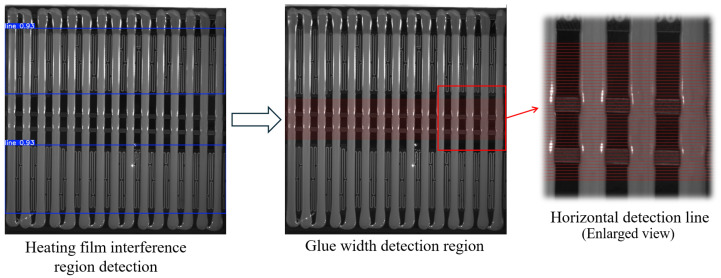
Schematic diagram of glue width detection region identification with two heating film interference regions.

For cases with multiple heating film interference regions, determining the measurement region becomes more complex. First, YOLOv11 is used to detect all interference regions and obtain their bounding box coordinates. Then, the interference regions are sorted in ascending order by $y_{\min}$ (the upper boundary of each interference region), forming an interference region sequence $\left\{\left(y_{1_{1}},y_{2_{1}}\right),\left(y_{1_{2}},y_{2_{2}}\right),\ldots ,\left(y_{1_{k}},y_{2_{k}}\right)\right\}$, where *k* represents the number of interference regions. Next, the gaps between adjacent interference regions are calculated, i.e., $\left[y_{2_{i}},y_{1_{i+1}}\right]$ (where i=1,2,…,k−1, typically k=3). Since the detection intervals are relatively few in this case, 80% of the center of each gap is selected as the measurement region, and detection lines are chosen at intervals of 10 pixels for measuring the glue strip width, as shown in Figure 7.

### 3.6. Glue Strip Width Measurement

To accurately measure the width of the glue strips in the glue region and effectively overcome the impacts of image noise, pixelated aliasing effects, and uneven glue application, a glue strip width measurement method based on connected component labeling and multi-line statistical analysis is proposed. This method aims to reduce the influence of image acquisition noise, aliasing effects, and local fluctuations during the glue process on the measurement results, thereby ensuring higher precision and robustness in the measurements.

**Figure 8:**
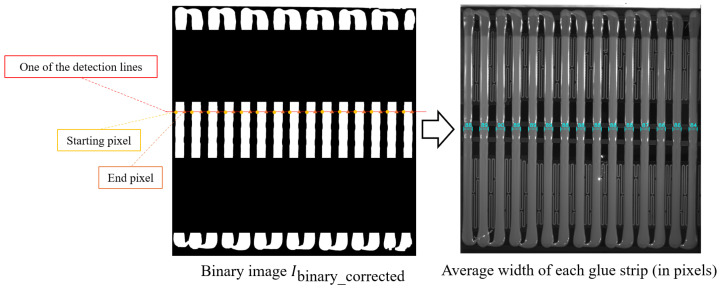
Schematic diagram of glue width calculation.

Specifically, the measurement of the glue strip width is performed by scanning the binary image *I*_binary_corrected_ row by row in the horizontal direction. In each row, the width of the detected glue strip is calculated based on the start and end pixel positions, and further averaged across multiple rows using statistical analysis to obtain the average width of each glue strip, as shown in Figure 8. The specific steps are as follows.

First, within the predefined measurement region, the binarized image is scanned row by row in the horizontal direction. For the *i*-th scan line, the white region of each detected glue strip in that line is determined by the starting pixel ${\mathrm{start}}_{j,i}$ and the ending pixel ${\mathrm{end}}_{j,i}$. The width $w_{j,i}$ is calculated using the following formula:

(5) $$w_{j,i}={\mathrm{end}}_{j,i}-{\mathrm{start}}_{j,i}$$

where $w_{j,i}$ is the width of the *j*-th glue strip in the *i*-th row.

For each glue strip *j*, the average width $W_{j}$ is computed by averaging the width values $w_{j,i}$ across all detection lines:

(6) $$W_{j}=\frac{1}{M_{j}}\sum_{i=1}^{M_{j}}w_{j,i}$$

where $M_{j}$ is the number of detection lines used to compute the average width for the *j*-th glue strip.

Finally, the overall average width $W_{\mathrm{avg}\;}$ of all glue strips is calculated:

(7) $$W_{\mathrm{avg}}=\frac{1}{C}\sum_{j=1}^{N}W_{j}$$

where *N* represents the total number of glue strips.

### 3.7. Glue Strip Length Measurement

**Figure 9:**
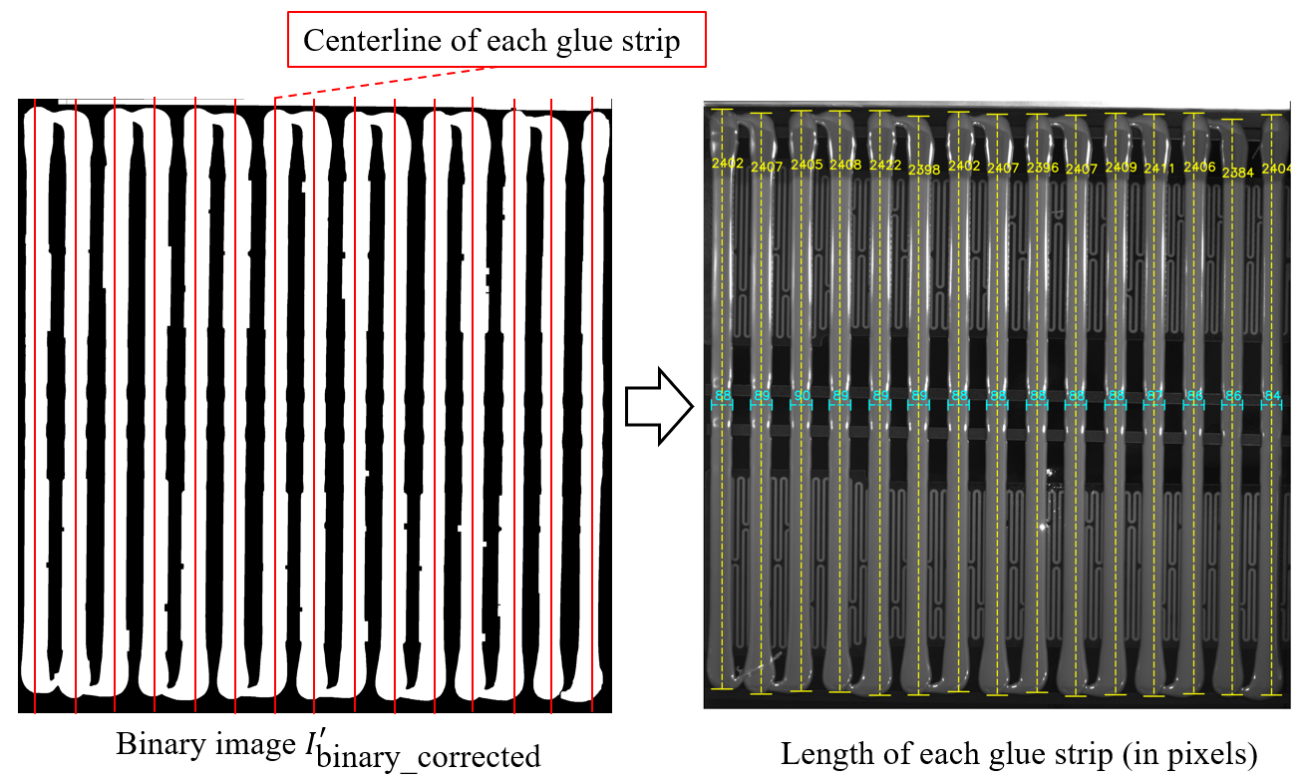
Schematic diagram of glue length calculation.

The measurement of glue strip length is primarily based on the midpoint of each glue strip’s width. The longest continuous white region is searched along the vertical direction, as shown in Figure 9. However, since the binarized image *I*_binary_corrected_ undergoes morphological closing operations, the glue head and the base edge are very close in the original image. This could cause the glue strip’s head to connect with the base edge in the binarized image, thereby affecting the accurate calculation of glue strip length. To solve this problem, we define a new binarized image $I_{\mathrm{binary\_corrected}}^{\prime }$ that is generated similarly to $I_{\mathrm{binary\_corrected}}$, but uses a smaller binarization threshold ($\tau^{\prime }=0.75P_{m}$) based on experiments. This better separates the glue head from the base (since the base edge is usually darker due to light blockage) and avoids the connectivity issues caused by morphological closing operations. We then follow these steps to calculate the length of each glue strip.

First, the midpoint ${\mathrm{mid}}_{j}$ of the width of each glue strip *j* is calculated as

(8) $${\mathrm{mid}}_{j}=\frac{{\mathrm{start}}_{j}+{\mathrm{end}}_{j}}{2}$$

where ${\mathrm{start}}_{j}$ and ${\mathrm{end}}_{j}$ are the average starting and ending positions.

Next, starting from ${\mathrm{mid}}_{j}$, we scan $I_{\mathrm{binary\_corrected}}^{\prime }$ in the vertical direction and record the length $L_{j}$ of the longest continuous white region:

(9) $$L_{j}=\max\left(\left|\left\{i|I_{\mathrm{binary\_corrected}}^{\prime }\left(i,{\mathrm{mid}}_{j}\right)=1\right\}\right|\right)$$

where $I_{\mathrm{binary\_corrected}}^{\prime }\left(i,{\mathrm{mid}}_{j}\right)=1$ indicates a white pixel at position $\left(i,{\mathrm{mid}}_{j}\right)$, and |·| represents the count of continuous white pixels.

Finally, the average length $L_{\mathrm{avg}}$ of all glue strips is calculated as

(10) $$L_{\mathrm{avg}}=\frac{1}{N}\sum_{j=1}^{N}L_{j}$$

where $L_{j}$ is the length of the *j*-th glue strip.

### 3.8. Glue Strip Breakage Detection

**Figure 10:**
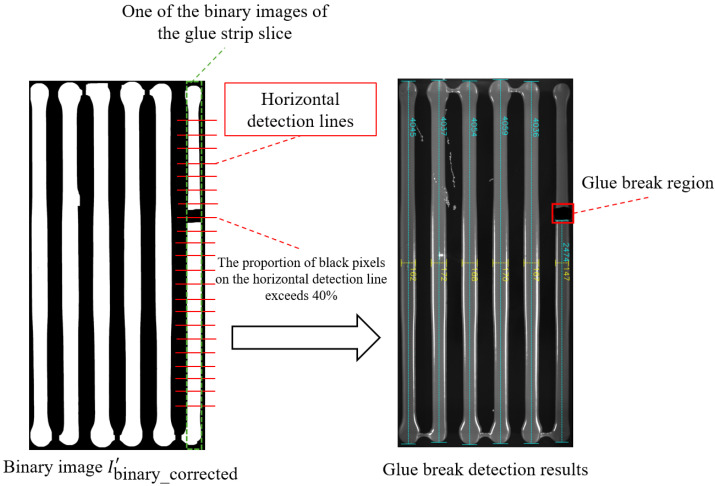
Schematic diagram of glue breakage detection.

The glue-breaking problem encompasses two primary phenomena: complete glue breakage and glue stringing. These defects are typically sporadic and occur with low frequency, resulting in a limited number of available samples. Consequently, deep learning-based detection networks trained on such small datasets are not suitable for reliably identifying these glue-breaking conditions. To address this challenge, this paper proposes a robust detection method based on image slicing and pixel proportion analysis. The proposed approach effectively overcomes the small-sample limitation and achieves accurate identification of glue-breaking defects, as demonstrated in Figure 10. The detailed procedure is described as follows.

First, calculate the average starting and ending positions for each glue strip *j*:

(11) $${\mathrm{starta}}_{{\mathrm{avg}}_{j}}=\frac{1}{M_{j}}\sum_{i=1}^{M_{j}}{\mathrm{start}}_{j,i},{\mathrm{end}}_{{\mathrm{avg}}_{j}}=\frac{1}{M_{j}}\sum_{i=1}^{M_{j}}{\mathrm{end}}_{j,i}$$

where $M_{j}$ is the number of samples for the *j*-th glue strip, and ${\mathrm{start}}_{j,i}$ and ${\mathrm{end}}_{j,i}$ represent the starting and ending positions of the *i*-th sample, respectively.

Based on these average positions, the binarized image $I_{\mathrm{binary\_corrected}}^{\prime }$ is sliced vertically, generating sub-images with a width of end _avg_*j*__ − start _avg_*j*__. Next, for each slice, the black pixel ratio $p_{j,i}$ in the central 70% region (avoiding the first and last 15% of the region) of each horizontal line *i* is calculated:

(12) $$p_{j,i}=\frac{{\mathrm{black}}_{j,i}}{{\mathrm{total}}_{j,i}}$$

where ${\mathrm{black}}_{j,i}$ is the number of black pixels on the *i*-th horizontal line, and ${\mathrm{total}}_{j,i}$ is the total number of pixels on that horizontal line.

In our implementation, if $p_{j,i}<40\%$, the location is considered a glue breakage region, and this position is marked. Finally, all detection information is annotated on the original image, including the width of each glue strip, average glue width, the length of each glue strip, average glue length, and glue breakage regions.

## 4. Experiment and Results Analysis

To comprehensively evaluate the effectiveness of the proposed battery pack glue detection method based on YOLOv11 and subpixel geometric analysis, a series of experiments were designed and conducted. The experiments were performed on a dataset containing 200 real production-line battery pack glue images. This dataset includes images from various vehicle models, different lighting conditions, and complex scenarios involving glue breakage, stringing, and heating film interference. The training process for all YOLO models was carried out on a server equipped with an Intel(R) Core(TM) i9-12900K processor (12th generation), 64GB of RAM, and an NVIDIA GeForce RTX 3060 graphics card. All evaluation experiments were conducted on an industrial control computer with an Intel Core i3-12100 processor. The software environment for these experiments was Python 3.9 and PyTorch 2.0.

### 4.1. YOLOv11-Based Object Detection

In this experiment, we employ the YOLOv11 model for two main tasks: glue region detection and heating film interference region detection. To evaluate the performance of YOLOv11 in these tasks, we conduct multiple experiments, each providing detailed evaluation data.

Firstly, in the glue region detection experiment, we used a custom battery pack glue dataset, which was augmented through data augmentation techniques. The training set consisted of 167 original annotated images, which were expanded to approximately 800 images using random flips, rotations, and other methods to increase data diversity. During training, we also prepared an independent test set to verify the model’s generalization ability. The test set consisted of 100 unseen images, which were used to evaluate the performance of YOLOv11 in glue region detection. The model training lasted for 12 h.

**Figure 11:**
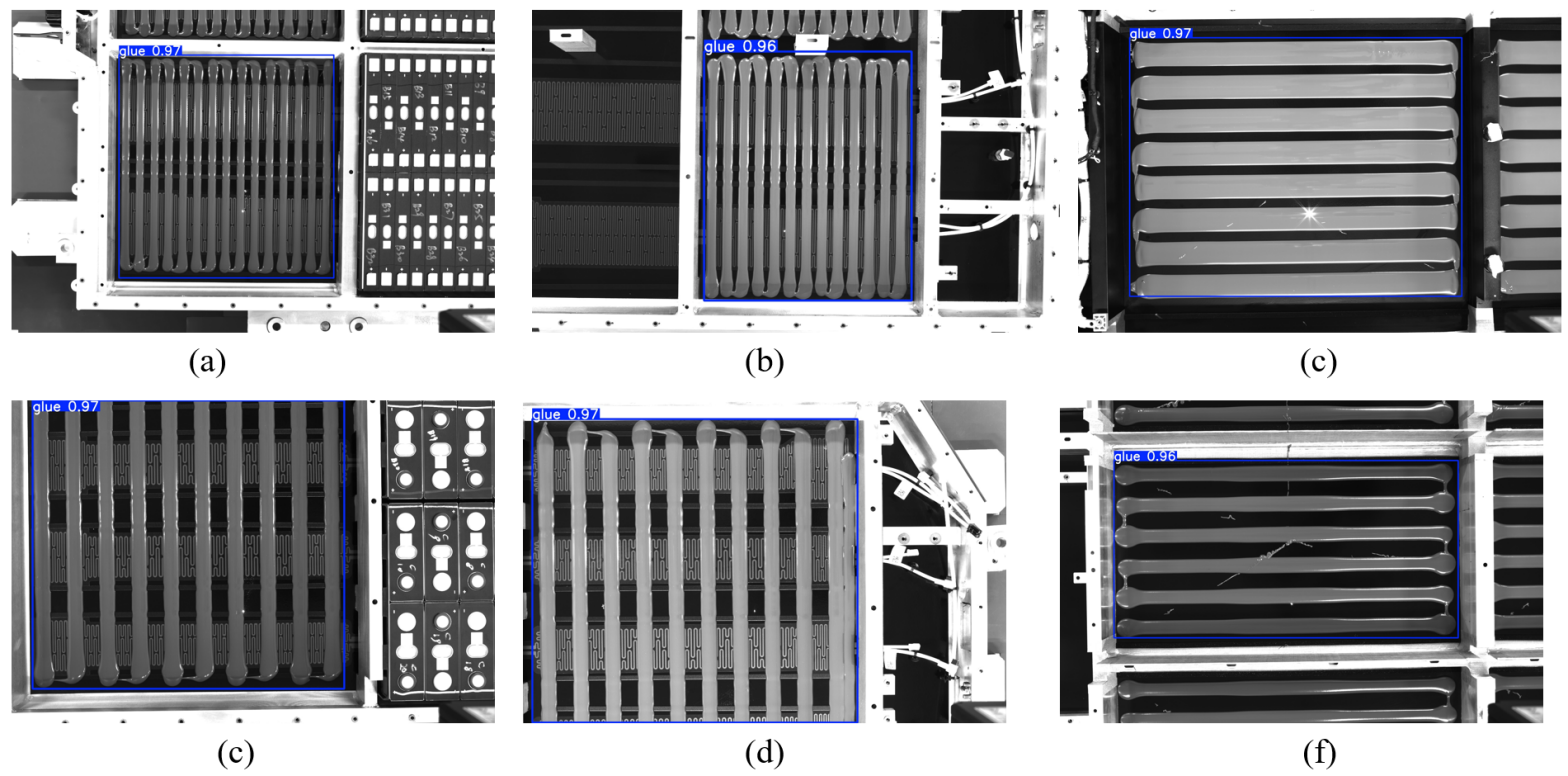
Glue region detection experiment results. (**a**–**f**) represent partial experimental results from the samples.

Qualitative results are shown in Figure 11. The experiment demonstrates that YOLOv11 accurately extracts glue regions even in complex backgrounds. In the quantitative analysis, we use the standard mean Average Precision (mAP) in object detection as the primary evaluation metric. The experimental results show that the model achieves an mAP@0.5 of 97.8% at an IoU threshold of 0.5, an mAP@0.75 of 96.3% at an IoU threshold of 0.75, and an mAP@0.5:0.95 of 97.1% at a stricter IoU threshold range of 0.5:0.95. These results demonstrate the model’s excellent performance in robustness and detection accuracy. In terms of inference time, the average inference time on the test set is 0.8 s per image. These results indicate that the trained model not only performs excellently in detection accuracy but also meets the practical application requirements in terms of inference speed.

In the heating film interference region detection experiment, we similarly used the augmented custom battery pack glue dataset for training. The training set consisted of 167 original annotated images, which were expanded to approximately 800 images using random flips, rotations, and other data augmentation techniques. As with glue region detection, an independent test set containing 100 unseen images was used to assess the performance of YOLOv11 in heating film interference detection. The training process lasted for 12 h.

**Figure 12:**
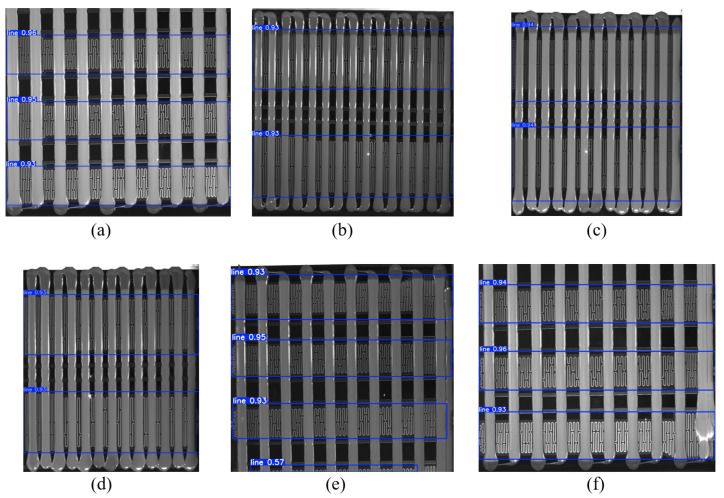
Heating film region detection experiment results. (**a**–**f**) represent partial experimental results from the samples.

Qualitative results are shown in Figure 12, which indicates that the model can effectively detect heating film interference regions. In the quantitative analysis, the mAP@0.5 reaches 95.7%, the mAP@0.75 is 94.4%, and the overall mAP@0.5:0.95 under the stricter IoU threshold range of 0.5:0.95 is 94.3%, highlighting the high precision of the model in locating and recognizing heating film regions. For inference time, YOLOv11 takes an average of 1.1 s per image for heating film region detection. The combination of both qualitative and quantitative results demonstrates that the model can accurately recognize heating film interference regions and exhibit strong adaptability in complex backgrounds.

### 4.2. YOLOv11-Seg-Based Glue Strip Orientation Detection and Adaptive Binarization Threshold

In our method, the YOLOv11-seg model is used to calculate the glue strip orientation and the binarization threshold. In this section, we first evaluate the performance of YOLOv11-seg in glue strip detection. Similar to the previous experiments, we used a data-augmented custom battery pack glue dataset for training. The training set consisted of 167 original mask-labeled images, which were expanded to approximately 800 images through random flips, rotations, and other augmentation techniques to enhance data diversity. During the training process, we also prepared an independent test set to verify the model’s generalization ability. The test set consisted of 100 unseen images, which were used to assess the performance of the YOLOv11-seg model in glue strip segmentation. The model was trained for 12 h.

**Figure 13:**
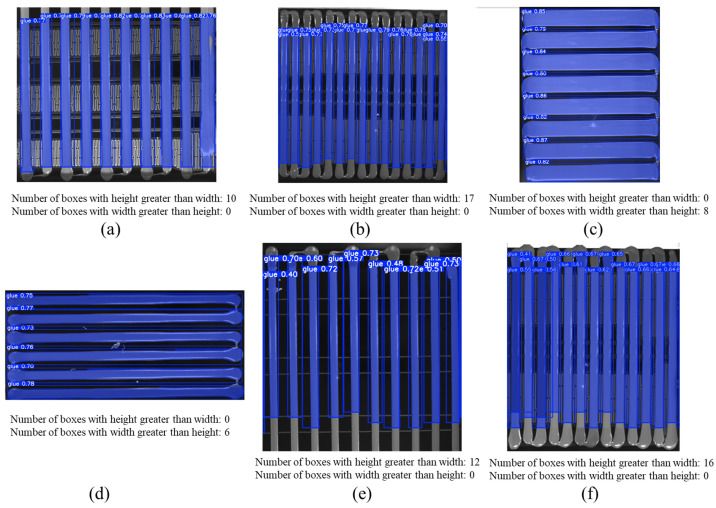
Glue strip instance segmentation experiment results. (**a**–**f**) represent partial experimental results from the samples.

The YOLOv11-seg model outputs both segmentation masks and bounding boxes. The average IoU for glue strip instance segmentation is 54.3%. The accuracy of the bounding boxes is measured with the standard metric mAP@0.5, which reaches 55.7%, mAP@0.75 at 54.4%, and mAP@0.5:0.95 at 54.3%. Qualitative segmentation results are shown in Figure 13. As observed, the model performs well in simple backgrounds (as shown in Figure 13c,d), but struggles with smaller glue strip targets and more complex backgrounds, particularly in the detection of the glue strip head region. In these cases, the model’s confidence in detecting the glue strip is lower, as shown in Figure 13b,e,f. The segmentation masks are incomplete, and the number of detected strips is incorrect.

For glue strip orientation detection in this method, we only reference the height-to-width ratio of the bounding boxes output by YOLOv11-seg. For example, in Figure 13b, a total of 17 bounding boxes are detected (with 15 actual glue strips). All 17 boxes have a height greater than width, so the glue orientation is judged as vertical, and no further processing is required. In contrast, in Figure 13c, 8 bounding boxes are detected, but none of them have a height greater than width, so the glue orientation is considered horizontal, and we rotate it 90° for further processing.

**Figure 14:**
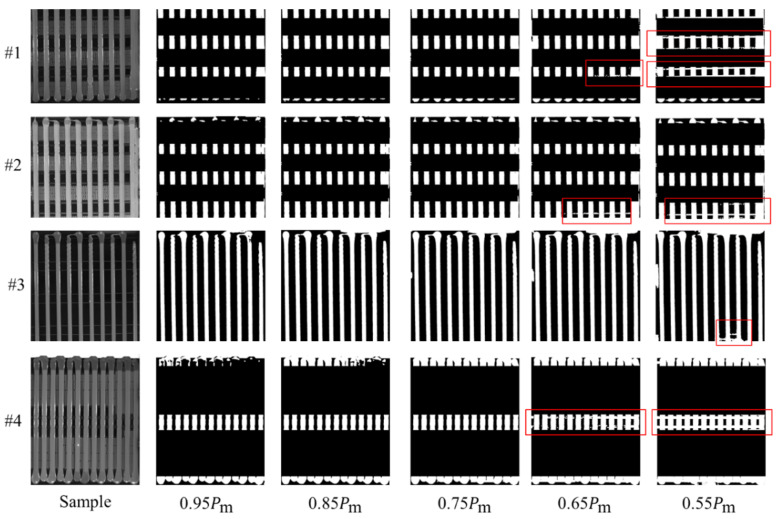
Adaptive threshold binarized image used for glue strip width measurement. When interfering regions caused by the heating film are detected, these regions are masked out. The red solid-line boxes indicate areas where the background and glue strips are difficult to distinguish due to an excessively low threshold.

In our method, both the width and length measurements of glue strips employ an adaptive binarization threshold. Specifically, the mean pixel intensity $P_{m}$ within the segmentation mask region produced by the YOLOv11-seg model is used as the reference value for determining the binarization threshold. Figure 14 illustrates the effectiveness of different adaptive binarization thresholds on glue strip width measurement. For width measurement, the primary objective is to ensure clear separation between the glue strip region and the background. If the threshold is set too low, background noise may be erroneously classified as foreground, causing adhesion between the glue strip and surrounding background regions (as highlighted by the red solid-line boxes in Figure 14). This significantly compromises the accuracy of subsequent width measurements. Experimental results demonstrate that binarization performance remains stable and robust when the threshold exceeds $0.75P_{m}$. To ensure measurement reliability while preventing excessive threshold values from eroding the actual glue strip areas, a threshold of $0.85P_{m}$ is selected as the adaptive binarization threshold for glue strip width measurement.

**Figure 15:**
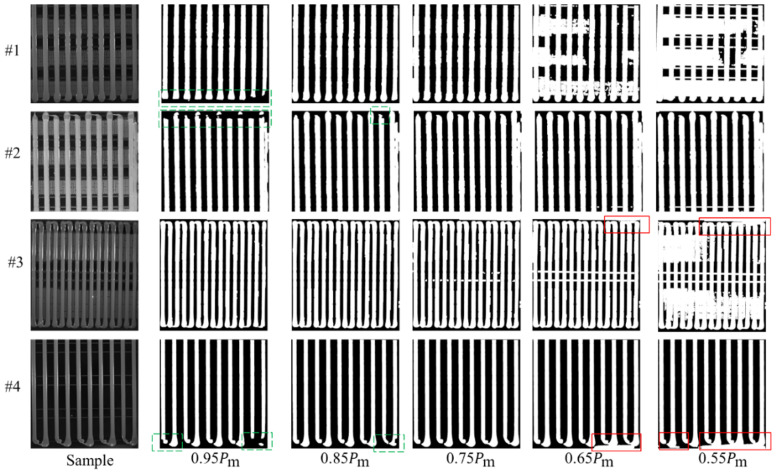
The adaptive threshold binarized image for glue strip length measurement. The area marked by the red solid-line box indicates where the glue strip merges with the image edge due to the threshold being set too low. The area marked by the green dashed-line box shows where the glue strip experiences breakage due to the threshold being set too high.

Figure 15 illustrates the impact of different adaptive binarization thresholds on the measurement of glue strip length. In length measurement, the main objective of automatically setting the binarization threshold is to prevent background erosion along the vertical measurement line at the center of the glue strip while ensuring effective separation of the edges from the background. When the threshold is set too low, it can cause the glue strip to merge with the background, as indicated by the red solid-line box in Figure 15. Conversely, when the threshold is set too high, the edge may appear darker under certain lighting conditions, leading to breakage of the glue strip region, as shown in the green dashed-line box in Figure 15. The experimental results indicate that setting the threshold close to $0.75P_{m}$ yields a more stable binarization effect. Therefore, in the glue strip length measurement process, we select $0.75P_{m}$ as the adaptive binarization threshold.

### 4.3. Glue Width and Length Measurement Evaluation

**Figure 16:**
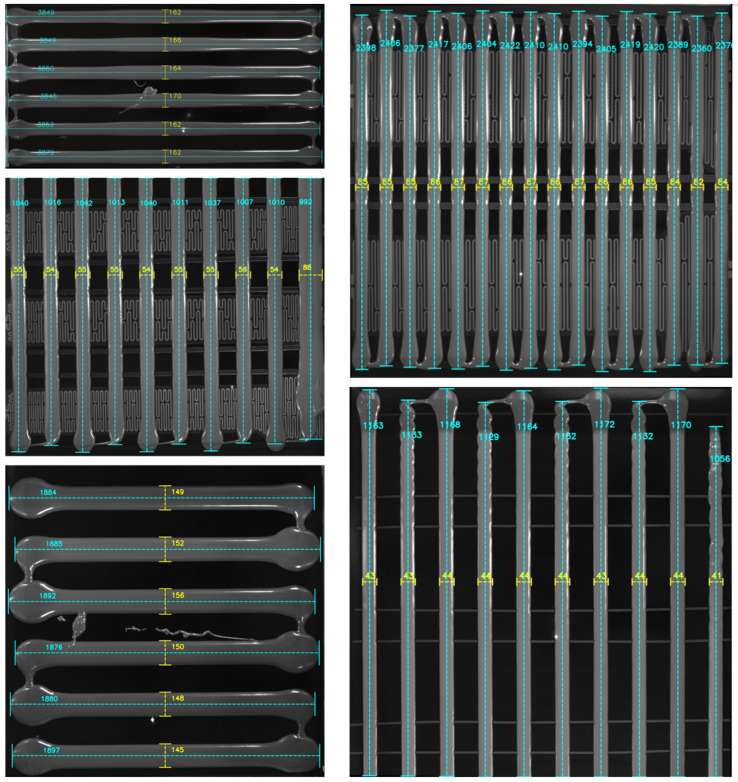
Glue strip length and width measurement results.

Figure 16 shows the measurement results of the length and width of each glue strip in the glue regions. Since the shooting positions vary for different vehicle models, the ratio between pixel values and actual lengths also differs. Therefore, we represent the glue strip length and width in terms of pixel values for consistency. The experimental results indicate that the proposed method can reliably identify the width and length of each glue strip.

In the quantitative analysis, we use the masks of each glue strip labeled during the training of the YOLOv11-seg model to calculate the real values of the glue strip length and width. Specifically, we first standardize the glue strip orientation to vertical, then calculate the real length by measuring the mask in the vertical direction. For the real width, we measure the average width in the center 70% of the glue strip.

To further validate the effectiveness of the proposed method in calculating the glue strip width and length, we compare it with traditional visual measurement methods and mainstream instance segmentation methods. For traditional visual measurement methods, we use the Canny edge detection algorithm to extract the edges of the glue strips, then calculate the glue strip width by counting the intersection points along horizontal measurement lines and determine the glue strip length by recording the maximum value along vertical measurement lines. Mainstream instance segmentation methods, on the other hand, directly extract the glue strip mask from the image and calculate the width and length based on this mask. Since the glue strip thickness and camera height vary across different vehicle models, we use error percentage and computation time as evaluation metrics. The comparison results of the different methods are shown in Table 1. The experimental results show that, although our method has a slightly increased computation time, it remains within an acceptable range and significantly outperforms other methods in measurement accuracy, with the error meeting the requirements of the glue detection process.

### 4.4. Glue Breakage Detection Evaluation

**Figure 17:**
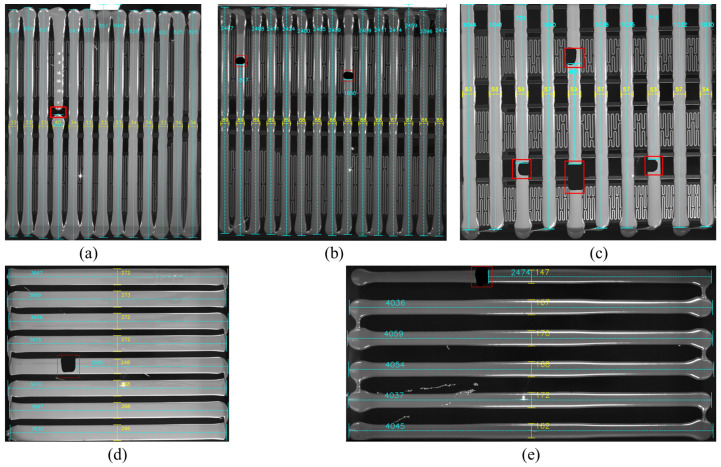
Glue strip breakage detection results. Red rectangular boxes indicate breakage regions. (**a**–**e**) represent partial experimental results from the samples.

Due to the limited number of breakage samples in the dataset, we manually created some breakage scenarios (such as glue strip breakage or stretching). Figure 17 shows the detection results of our method in different breakage cases, where Figure 17a represents a real breakage scenario, and the rest are artificially created breakage situations. The experimental results indicate that our method can reliably and accurately identify breakage regions, achieving a detection rate of 100%, and is not affected by complex backgrounds. It should be noted that although the longest connected part length of the glue strip is still marked in the figure, we have excluded the data of broken glue strips when calculating the average glue strip length to ensure the stability of the calculation results.

### 4.5. Restoration of Original Image with Detection Information

**Figure 18:**
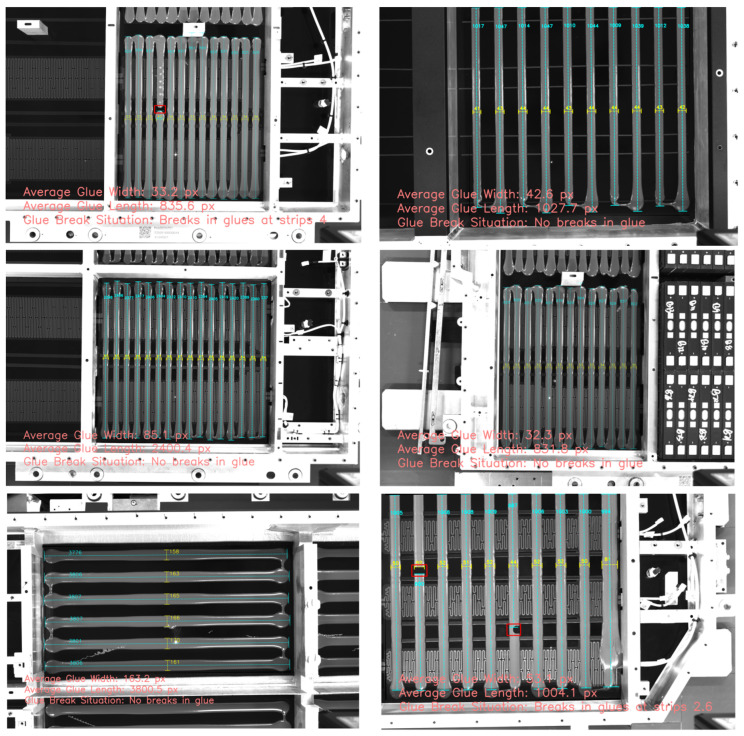
Comprehensive annotation of glue application detection information. Red rectangular boxes indicate breakage regions.

After completing the measurement of glue strip width, length, and breakage detection, we restore the annotated glue region images using the inverse of the rotation matrix and overlay the restored images back onto the original images. The final result is the original image with detection annotations, as shown in Figure 18. Workshop personnel can directly use these annotations to assess whether the glue application process meets the required standards, or provide the relevant data to the PLC system to determine if adjustments to the glue application robotic arm are necessary.

### 4.6. Ablation Experiment

To comprehensively validate the effectiveness of the proposed method and the contribution of each key module, we design and conduct a systematic ablation experiment on the same test set. This experiment focuses on evaluating the combined impact of the following three core modules on the accuracy of glue strip width and length measurements: Module A: Glue strip orientation fine correction module; Module B: Adaptive binarization threshold module; Module C: Dynamic glue width detection line region identification. The experimental results for each module combination are shown in Table 2.

As shown in Table 2, the impact of glue strip orientation fine correction on the measurement results is relatively small. This is mainly because, in industrial production lines, the camera installation position is relatively fixed, and the glue strips captured in the original images are mostly aligned either horizontally or vertically. Therefore, the projection errors eliminated by geometric rotation correction are relatively minor. Nevertheless, this module still provides a unified geometric reference for subsequent high-precision measurements. The introduction of Module B (Exp. 3) or Module C (Exp. 4) significantly reduces the measurement errors. In particular, Module B reduces the glue width error from 21.4% to 10.4%. This is due to the differences in the surface material, reflective properties, and lighting conditions of battery packs across different vehicle models, which make it difficult for fixed thresholds to adapt to diverse detection environments. The adaptive threshold strategy effectively overcomes the segmentation challenges caused by uneven lighting. Module C, on the other hand, uses dynamic region detection to effectively avoid noise from non-glue regions, especially addressing complex texture interference such as heating film lines, thereby preventing significant impact on the glue width calculation. When all three modules are integrated (Exp. 8), the system achieves the best performance, with a glue width error of only 1.5% and a glue length error of 2.3%. This indicates that the modules complement each other well and work together to ensure robust and high-precision measurements in complex industrial environments.

## 5. Conclusions

This paper proposes a battery pack glue detection method based on YOLOv11 and pixel geometric analysis, aimed at solving the problems of dimension measurement and defect detection in the glue process of new energy vehicle battery packs in complex environments. The method combines the YOLOv11 model for glue region extraction, while interference suppression and uniform glue strip orientation representation significantly improve the accuracy and consistency of the glue region. Through adaptive binarization, morphological processing, and Hough transform correction, the detection accuracy of the glue region is further enhanced. The introduced pixel geometric analysis, along with image slicing and pixel ratio analysis mechanisms, not only allows for precise measurement of the glue strip’s width and length but also effectively detects defects such as glue breakage and stringing. Even in complex backgrounds and environmental interferences, the method remains stable in achieving high-precision glue dimension measurements and demonstrates outstanding robustness in glue strip breakage detection. Compared to traditional visual measurement methods and mainstream deep learning models, the proposed method significantly reduces measurement errors, with a glue width error of only 1.5% and a glue length error of 2.3%, far surpassing existing methods. The method exhibits good adaptability and stability in handling issues such as heating film interference, glue strip orientation and layout differences across vehicle models.

Additionally, the ablation experiment results further validate the contribution of each key module to the system’s performance. The experiments show that the integration of glue strip orientation fine correction, adaptive binarization threshold, and dynamic glue width detection line region identification modules significantly enhances the system’s accuracy and robustness. The proposed method provides an efficient, precise, and adaptable detection solution for the glue process of new energy vehicle battery packs, offering substantial industrial application value. Future work will focus on multi-modal fusion (e.g., incorporating 3D point cloud data) and cross-model generalized training to accommodate a wider range of battery pack design variations, advancing the glue detection toward zero-defect production.

## Figures and Tables

**Table 1:** Comparison of different methods in terms of glue strip width, length calculation, and runtime. The optimal results are highlighted in bold.

Method	Width Error (%)	Length Error (%)	Runtime (s)
Traditional visual measurement	59.2	10.1	3.4
Mask R-CNN	23.1	34.6	3.9
YOLOv9-seg	25.7	37.9	1.8
YOLOv10-seg	26.4	34.2	1.6
YOLOv11-seg	24.0	35.7	**1.5**
Ours	**1.5**	**2.3**	5.7

**Table 2:** Ablation study results of different module combinations. A, B, and C represent the glue strip orientation fine correction module, adaptive binarization threshold module, and dynamic glue width detection line region identification, respectively. “✓” indicates the module is used, and “×” indicates it is not used. The best results are highlighted in bold.

Exp.	A	B	C	Width Error (%)	Length Error (%)
1	×	×	×	21.4	26.7
2	✓	×	×	20.6	25.1
3	×	✓	×	10.4	9.8
4	×	×	✓	12.7	11.6
5	✓	✓	×	10.0	9.2
6	✓	×	✓	12.1	11.3
7	×	✓	✓	1.9	2.8
8	✓	✓	✓	**1.5**	**2.3**

## Data Availability

Dataset available on request from the authors.
